# Krüppel-like factor 4 regulates cellular proliferation and differentiation in human bone marrow-derived mesenchymal stem cells

**DOI:** 10.1016/j.bbrep.2025.102241

**Published:** 2025-09-06

**Authors:** Kenichi Miyamoto, Satoru Miyagi, Rintaro Yoshikawa, Yuichi Michikawa, Yumi Matsuzaki

**Affiliations:** a Department of Life Science, Faculty of Medicine, Shimane University, 89-1 Enya-cho, Izumo City, Shimane, 693-8501, Japan; b Department of Biochemistry, Faculty of Medicine, Shimane University, 89-1 Enya-cho, Izumo City, Shimane, 693-8501, Japan; c Human Resources and Networking Section, Department of Co-creation Promotion, Institute of Radiological Science, National Institute for Quantum Science and Technology (QST), Anagawa 4-9-1, Inage-ku, Chiba, 263-8555, Japan; d PuREC Co., Ltd., 89-1 Enya-cho, Izumo City, Shimane, 693-0021, Japan

**Keywords:** Mesenchymal stem cells, Krüppel-like factor 4, Proliferation, Differentiation

## Abstract

Mesenchymal stem cells (MSCs) form one of the types of adult stem cell which have the capacity to self-renew, and the multipotentiality for osteogenic, chondrogenic and adipogenic differentiation and immune modulation. Consequently, MSCs are an attractive cell source for regenerative medicine and therapy for inflammatory disease. However, biological criteria to ensure any particular MSCs are “stem cells” have not been clarified. Previously, we reported that MSCs isolated from a single CD90/CD271 double-positive cell in human bone marrow have high colony-forming capacity and tri-lineage differentiation potential *in vitro*. Such clonal MSCs are highly homogeneous and considered to be useful for the analysis of molecular function. In this study, we focused on Krüppel-like factor 4 (KLF4) which is highly expressed by our MSC subtype, and examined its role *in vitro*. The expression of KLF4 was significantly reduced within 24 h after the induction of adipogenic or osteogenic differentiation. Knockdown of KLF4 led to promotion of cellular proliferation and differentiation at an early stage. Furthermore, the expressions of *TGFBR1*, *FZD6*, *FGFR2*, *THY1* and *CXCL12* genes were upregulated in KLF4 knockdown MSCs. These results indicated that KLF4 regulates not only cellular proliferation but also the early stage of differentiation. Our findings suggest that KLF4 has an important role in maintaining the properties of MSCs and regulating differentiation via TGF-β, WNT and FGF signaling pathways.

## Highlights

• KLF4 is highly expressed in MEC, a subtype of our MSCs.
• KLF4 regulates cellular proliferation and early stages of differentiation.
• KLF4 affects expression of genes encoding TGF-β, WNT and FGF signaling receptors.

## 1 Introduction

Mesenchymal stem cells (MSCs) are adult stem cells isolated from bone marrow, adipose tissue, dental pulp or umbilical cord tissues, with characteristics of plastic adherence, fibroblastic morphology, absence of hematopoietic markers and the capacity for adipogenic, osteogenic and chondrogenic differentiation [1,2]. MSCs are also reported to possess immunosuppressive properties and therefore they are an attractive cell source for regenerative medicine and therapy for immune diseases (e.g., graft versus host disease, Crohn’s disease) [3,4]. However, conventionally-established MSCs, as described by Pittenger and colleagues, are a heterogeneous cell population because the selection method relies simply on plastic adherence, although the clonal complexity is subsequently reduced with the increase in the number of passages [5].

We previously reported that prospective isolation of CD90 and CD271 double-positive cells from human bone marrow enabled us to establish highly-proliferative MSC clones [6,7]. Three subtypes of MSCs, REC (Rapidly Expanding MSC Clone), MEC (Moderately Expanding MSC Clone) and SEC (Slowly Expanding MSC Clone), which differ in their proliferation rate, are homogeneous cells due to their expansion from a single original cell, and each of the clones has tri-lineage differentiation ability. Therefore, we consider that they are more advantageous than conventional MSCs for the analysis of molecular function.

Krüppel-like factors (KLFs) are transcription factors containing a zinc finger motif at the C-terminus, and function as transcriptional activators or repressors by binding to the CACCC sequence or GT box on DNA elements [8]. KLF4 is a well-known member of the KLF family and plays critical roles in maintaining physiological functions in tissues and organs. In stem cells, KLF4 possesses the pivotal role in stemness maintenance of pluripotent stem cells and serves to repair tissue injury in intestinal stem cells and hair follicle stem cells [9]. However, its function in MSCs is poorly understood. In this study, we investigated the role of KLF4 in human bone marrow-derived MSCs *in vitro* using our highly homogeneous MSC clones.

## 2 Materials and methods

### 2.1 MSC isolation and cell culture

Human bone marrow-derived MSC clones, named REC, MEC and SEC, were isolated and expanded as described previously [7]. Briefly, a vial of frozen human bone marrow mononuclear cells purchased from Lonza Bioscience (Walkersville, MD, USA) was thawed in a 37 °C water bath for 1 min then the cells were resuspended in HBSS + medium supplemented with 2 % fetal bovine serum (FBS; Hyclone, Logan, UT, USA) and 1 % penicillin/streptomycin. After washing with Hanks’ balanced salt solution (HBSS+), the cells were resuspended in HBSS+ and stained with anti-human CD90-APC antibody (BD Biosciences, Franklin Lakes, NJ, USA) and anti-human CD271-PE antibody (Miltenyi Biotec, Auburn, CA, USA) and propidium iodide. Single CD90/CD271 double-positive live cells were sorted into individual wells of a 96-well plate using a Moflo XDP cell sorter (Beckman Coulter, Brea, CA, USA). After culture for 2–4 weeks, colonies more than 90 % confluent at 2 weeks were harvested and classified as REC, colonies confluent at 3 weeks were classified as MEC and the other clones less than 50 % confluent at 4 weeks were harvested and classified as SEC. Cells were cultured in Dulbecco’s modified Eagle medium (DMEM) (Fujifilm Wako Pure Chemical Industries Ltd., Osaka, Japan) supplemented with 20 % FBS, 10 mM HEPES (Fujifilm Wako Pure Chemical Corporation), 1 % Penicillin/Streptomycin (Fujifilm Wako Pure Chemical Corporation) and 20 ng/mL basic fibroblast growth factor (bFGF) (Fujifilm Wako Pure Chemical Corporation). Established MSC clones were provided by PuREC Co., Ltd.

### 2.2 Induction of adipogenic and osteogenic differentiation

Cells were seeded at 3 × 10^4^/well into wells of a 24-well plate and next day, the medium was replaced with adipogenic differentiation medium (growth medium without bFGF supplemented with 1 μM Dexamethasone, 200 μM Indomethacin and 0.5 mM IBMX (isobutyl methylxanthine)) and cultured for 2 weeks. To induce osteogenic differentiation, the medium was supplemented with 0.1 μM Dexamethasone, 10 mM β-glycerophosphate and 50 μM ascorbic acid and cells were cultured for 3 weeks. In each case the medium was changed twice per week.

### 2.3 Oil Red O staining

After adipogenic induction, cells were fixed with 4 % PFA for 15 min at room temperature (RT). They were then washed with PBS, rinsed with 60 % isopropanol and stained with Oil red O staining solution (Muto Pure Chemicals, Tokyo, Japan) for 30 min at RT. After staining, Oil red O was extracted in isopropanol and the absorbance of the collected dye was measured at 490 nm using a DTX880 multi-mode plate reader (Beckman Coulter).

### 2.4 Alizarin Red S staining

After osteogenic induction, cells were fixed with 4 % PFA for 15 min at RT, washed with PBS, then stained with Alizarin red S (Muto Pure Chemicals) for 30 min at RT. Alizarin red S was extracted using 10 % acetic acid and neutralized with 10 % ammonium hydroxide. The absorbance of the extracted dye was measured at 405 nm using a DTX880 multi-mode plate reader.

### 2.5 Plasmid construction

Short-hairpin RNA (shRNA) was designed using BLOCK-iT RNAi Designer (Thermo Fisher Scientific, Waltham, MA, USA). Annealed oligos were ligated with the pENTR4-H1 plasmid at the BglII/XbaI site. The resulting plasmids were recombined with CSII-RfA-EG lentiviral plasmid using Gateway LR clonase II enzyme mix (Thermo Fisher Scientific). pENTR4-H1 and CSII-RfA-EG plasmids were obtained from RIKEN BioResource Center (Tsukuba, Japan). The target sequences of KLF4 were 5’-GGACGGCTGTGGATGGAAATT-3’ for shKLF4_#1, and 5’-GCACTACAATCATGGTCAAGT-3’ for shKLF4_#2. The non-specific target sequence as scrambled control was 5’-AAGGGATTTACATGGTTTAT-3’.

### 2.6 Production and transduction of lentivirus

Lenti-X 293T cells (Takara Bio, Shiga, Japan) were transfected with lentiviral plasmid and packaging plasmids, pCAG-HIVgp and pCMV-VSV-G_RSV-Rev (RIKEN BioResource Center) using polyethyleneimine “MAX” (Polysciences Inc., Warrington, PA, USA). Viral supernatant was collected at 48 h and 72 h after transfection. Collected lentivirus was concentrated by centrifugation at 6,000×*g* at 4 °C for 16 h and then stored at −80 °C until use. For lentiviral transduction, 1 × 10^5^ cells of MEC were seeded into wells of a 6-well plate and lentivirus was transduced in the presence of 50 μg/mL protamine sulfate for 48 h. Infected GFP-positive cells were sorted using a MoFlo XDP cell sorter (Beckman Coulter).

### 2.7 Quantitative RT-PCR (qRT-PCR)

Total RNA was extracted using RNAiso plus (Takara) and treated with RNase free DNase I (Roche, Basel, Switzerland). Reverse transcription was performed using PrimeScript II Reverse Transcriptase (Takara) according to the manufacturer’s protocol. Quantitative RT-PCR was performed using Fast SYBR Green Master Mix and a 7500 FAST real time PCR system (Thermo Fisher Scientific). Expression of each gene was normalized to the expression of *EEF1A1*. All assays were run in triplicate. Primer sequences used in assays are listed in Supplemental Table 1.

### 2.8 Immunoblotting

Protein samples were prepared by lysing the cells with 1 × Laemmli sample buffer. Samples were subjected to sodium dodecylsulfate polyacrylamide gel electrophoresis (SDS-PAGE) and transferred to a polyvinylidene difluoride (PVDF) membrane (Cytiva, Wilmington, DE, USA) by electroblotting. The transferred membrane was blocked with 5 % skimmed milk/TBST at RT for 1 h, then exposed to the primary antibody at RT for 1 h or at 4 °C overnight. After washing with TBST three times, the membrane was exposed to HRP-conjugated mouse or rabbit IgG at RT for 1 h, followed by another three washes in TBST, then a chemiluminescent reaction was detected using ECL™ prime (Cytiva) and LAS-4000 (FujiFilm) or ImageQuant800 (Cytiva). Intensities of chemiluminescence were measured using Fiji software [10]. Primary antibodies used in this study were: anti-KLF4 (ab215036, 1:1000 dilution; Abcam, Cambridge, MA, USA), anti-Lamin A/C (ab108922, 1:5000 dilution; Abcam), anti-TGFBR1 (ab31013, 1:1000 dilution; Abcam), anti-FGFR2 (ab109372, 1:1000 dilution; Abcam), and anti-FZD6 (#5158, 1:1000 dilution; Cell Signaling Technology, Beverly, MA, USA).

### 2.9 Microarray and data analysis

Microarray analysis was performed using the Human Genome U133 Plus 2.0 platform (Affymetrix, Thermo Fisher Scientific). Expression data were normalized using an RMA algorithm and compared by RankProd 2.0 using R software (version 4.4.0) [11]. Gene set enrichment analysis (GSEA) was performed using the software (version 4.2.3) provided by the Broad institute (https://www.gsea-msigdb.org/gsea/index.jsp). Normalized microarray data are available in Supplementary file 1.

### 2.10 Statistical analysis

All statistical analyses were performed using R software. Data for quantitative analyses were analyzed by Shapiro-Wilk test and Student’s t-test. Data are represented as the mean ± standard deviation.

## 3 Results

### 3.1 KLF4 is more highly expressed in MEC than in REC or SEC

To understand the genetic characteristics of our MSC subtypes, REC, MEC and SEC, transcriptome analysis using a microarray was performed and showed that the expressions of 31 genes were higher, while expressions of 14 genes were lower in MEC than REC (REC vs MEC, FDR <0.01) (Fig. 1A). Meanwhile the expressions of six genes were higher, and five genes were lower in MEC than in SEC (MEC vs SEC, FDR <0.01) (Fig. 1B). GSEA showed that gene sets involved with cell division were enriched in REC compared with MEC and in MEC compared with SEC (Fig. 1C and D). This observation reflects the fact that our MSC subtypes were distinguished by cellular proliferation rate as previously reported [6]. Among these differentially expressed genes, *KLF4* was highly expressed in MEC compared with REC and SEC. Quantitative RT-PCR confirmed that the expression of KLF4 was significantly higher in MEC compared with REC (approximately 7.6-fold for MEC/REC) and SEC (approximately 16.2-fold for MEC/SEC) (Fig. 1E). Conversely, no significant difference was observed between MEC and commercial bone marrow (BM)-MSCs (Fig. 1F). These results show that the expression of KLF4 is a characteristic feature of the MEC.

**Fig. 1:**
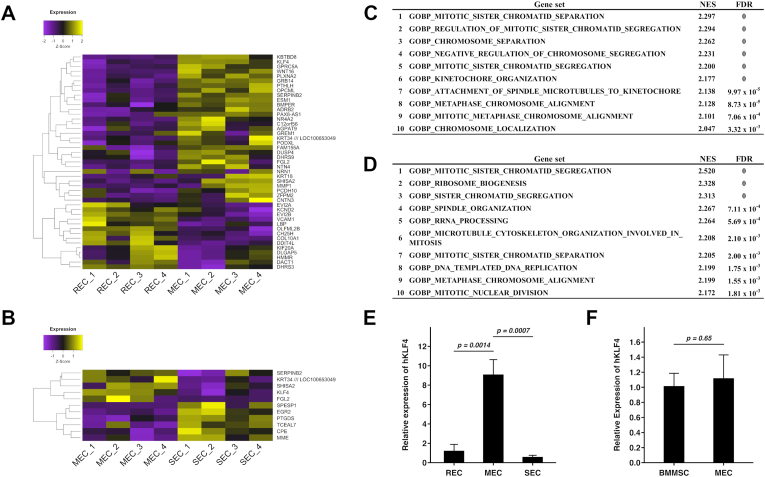
KLF4 is highly expressed in MEC compared with REC and SEC. (A and B) Heatmap of differentially expressed genes from microarray analysis (FDR <0.01) in REC compared with MEC and in MEC compared with SEC. (C) The top10 gene sets enriched in REC compared with MEC. (D) The top10 gene sets enriched in MEC compared with SEC. (E) Relative expression of *KLF4* in our MSC subtypes by qRT-PCR. (F) Relative expression of *KLF4* in the comparison of MEC with conventional MSCs by qRT-PCR. (E and F) Three independent experiments were performed using three independent MSC clones. Error bars represent standard deviation. NES: normalized enrichment score; FDR: false discovery rate.

### 3.2 Expression of KLF4 is reduced by induction of differentiation

Next, we examined whether expression of *KLF4* impacts the differentiation ability of MSCs. During adipogenic induction, expression of *KLF4* was observed in uninduced MEC (Day 0) but was markedly reduced at day 1 and reached a minimum at day 3 (Fig. 2A). Simultaneously, the adipogenic differentiation marker gene, *PPARG*, was upregulated (Fig. 2B). Similarly, marked reduction of *KLF4* expression was observed at day 1 after osteogenic induction and the osteogenic differentiation marker gene, *RUNX2*, was upregulated (Fig. 2C and D). In addition, a transient increase in KLF4 expression was observed at 3 h after adipogenic induction which gradually decreased thereafter up to 24 h, as shown by analysis of mRNA and protein levels (Fig. 2E and F). Similar trends were also observed during osteogenic induction (Fig. 2G and H). These data indicated that the expression of KLF4 was significantly decreased at 24 h after the induction of differentiation and suggested that KLF4 might be involved in the early stages of differentiation in MSCs.

**Fig. 2:**
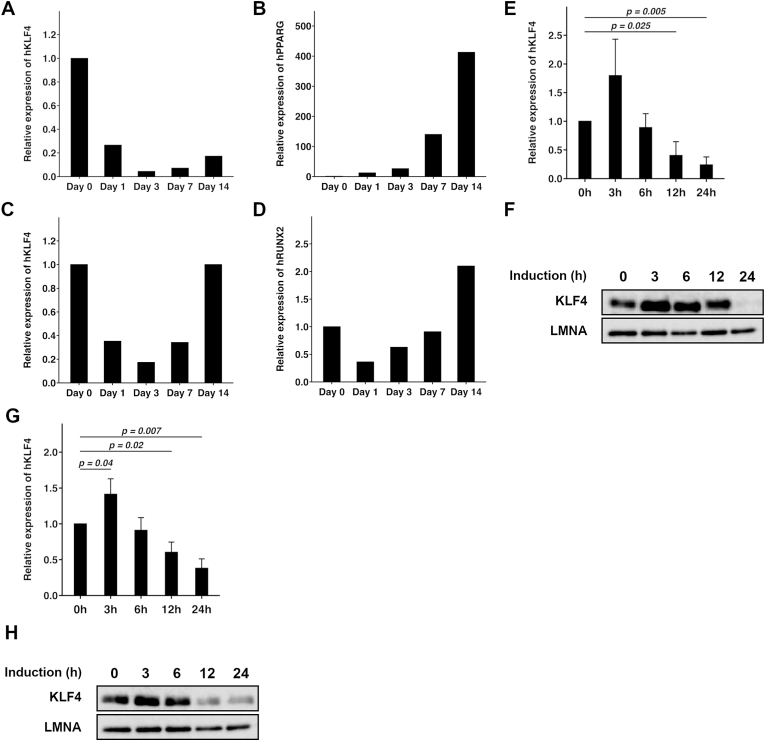
KLF4 is downregulated after induction of adipogenic or osteogenic differentiation. (A and B) Relative expression of h*KLF4* (A) and h*PPARG* (B) by qRT-PCR during adipogenic differentiation. (C and D) Relative expression of h*KLF4* (C) and h*RUNX2* (D) by qRT-PCR during osteogenic differentiation. (E) Relative expression of h*KLF4* by qRT-PCR at 24 h after induction of adipogenic differentiation. (F) Immunoblot of KLF4 at 24 h after induction of adipogenic differentiation. Lamin A (LMNA) was used as the internal control. (G) Relative expression of h*KLF4* by qRT-PCR 24 h after induction of osteogenic differentiation. (H) Immunoblot of KLF4 24 h after induction of osteogenic differentiation. Lamin A (LMNA) was used as the internal control. (E and G) Three independent experiments were performed using three independent MEC clones. Error bars represent standard deviations.

### 3.3 Knockdown of KLF4 promotes cellular proliferation and early differentiation

To further examine the association of KLF4 expression with MSC differentiation, KLF4 was knocked down in MEC by stable expression of shRNA (Fig. 3A and B). As shown in Fig. 3C, KLF4 knockdown (KLF4-KD) increased cellular proliferation compared with control. The phenotype induced by shKLF4_#1 was clearly stronger than that of shKLF4_#2 and therefore, shKLF4_#1 was used for subsequent assays. The increase in the proliferation rate was validated by the expression of *MKI67* which was significantly higher in KLF4-KD MEC than in control cells (Fig. 3D). In addition, we examined the differentiation capacity of KLF4-KD MEC. In adipogenic induction, the formation of lipid droplets in KLF4-KD MEC was significantly greater than that in control cells at day 7 but was comparable at day 14 (Fig. 3E and F). Similarly, in osteogenic induction, calcium deposition in KLF4-KD MEC tended to be higher than in control MEC at day 14 but was comparable at day 21 (Fig. 3G and H). These data show that decreased KLF4 promoted the early stages of both adipogenic and osteogenic differentiation.

**Fig. 3:**
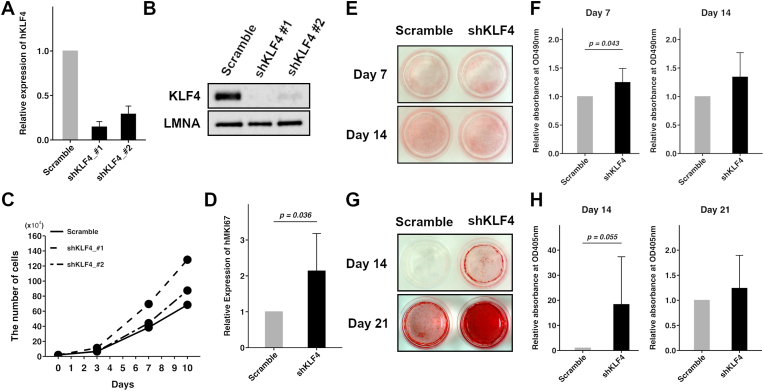
KLF4 knockdown affects cellular proliferation rate and differentiation in MEC. (A) Relative expression of h*KLF4* by qRT-PCR in KLF4-KD and control MECs. Scramble means non-specific gene-targeted shRNA control. Two independent shRNAs were designed and their efficiency examined using three independent MEC clones. (B) Immunoblot of KLF4 in KLF4-KD MEC. LMNA was used as the internal control. (C) Cellular proliferation rate of KLF4-KD MEC. Cells were counted at the indicated timepoints. Four independent experiments were performed using four independent clones. (D) Relative expression of h*MKI67* in KLF4-KD and control by qRT-PCR. Five independent experiments were performed using five independent MEC clones. (E) Oil Red O staining in KLF4-KD MEC and control at the indicated timepoints after induction of adipogenic differentiation. (F) Relative absorbance of Oil Red O in KLF4-KD MEC. Five independent experiments were performed using five independent MEC clones. Each experiment was run in duplicate. (G) Alizarin Red S staining in KLF4-KD MEC and controls at the indicated timepoints after induction of osteogenic differentiation. (H) Relative absorbance of Alizarin Red S in KLF4-KD MEC. Four independent experiments were performed using four independent MEC clones. Each experiment was run in duplicate. All quantitative data were normalized to those of each control cell. Error bars represent standard deviation.

### 3.4 KLF4 regulates the expression of signal receptor genes

Previously, Voutila and colleagues produced human MSCs overexpressing KLF4 by short activating RNA transfection or lentiviral transduction (KLF4-OE MSC), and analyzed their transcriptome [12]. To identify the genes affected by KLF4 overexpression, we reanalyzed their data and observed 27 upregulated genes and 76 downregulated genes in the KLF4-OE MSCs in comparison with control MSCs (Fig. 4A). In addition, GSEA showed that genes associated with autophagy were mainly enriched among the upregulated genes, and those associated with cell cycle regulation were enriched among the downregulated genes (Fig. 4B and C). This result agreed with the promotion of cellular proliferation in KLF4-KD MEC revealed in our data (Fig. 3C and D). Next, we selected some genes encoding signal receptors (*TGFBR1*, *FZD6* and *FGFR2*), cell surface molecules (*THY1*) and chemokine ligands (*CXCL12*) to ascertain whether the expressions of those genes were affected in KLF4-KD MEC (Supplemental Table 2). As expected, downregulated genes in KLF4-OE MSCs were significantly upregulated in KLF4-KD MEC with the exception of *CXCL12* (Fig. 4D–H). These observations indicate that KLF4-KD MEC shows, at least partially, an opposing transcriptome phenotype to that of KLF4-OE MSCs. Nonetheless, no significant increases of the signal receptor proteins, TGFBR1, FZD6 and FGFR2, were observed in KLF4-KD MEC (Fig. 4I–L). Finally, to investigate the association of the selected five genes with MSC differentiation, we examined their expression changes in adipogenic and osteogenic induction. Interestingly, the expressions of *TGFBR1* and *FZD6* were reduced under adipogenic induction but increased in osteogenic induction (Fig. 4M and N). On the other hand, the expression of *FGFR2* was low at day 0 but increased under both adipogenic and osteogenic induction (Fig. 4O). These results support the suggestion that TGF-β and WNT signal pathways acting via TGFBR1 and FZD6 contribute to osteogenic differentiation and that the FGF signal pathway acting via FGFR2 contributes to both adipogenic and osteogenic differentiation. Meanwhile, the expression of *THY1* was decreased under both induction conditions and *CXCL12* also decreased at day 1 under both adipogenic and osteogenic induction (Fig. 4P and Q). Thus, these results indicated that the expressions of these genes were changed with the downregulation of KLF4 during both adipogenic and osteogenic differentiation, and support the idea that KLF4 plays an important role in the differentiation of MSCs.

**Fig. 4:**
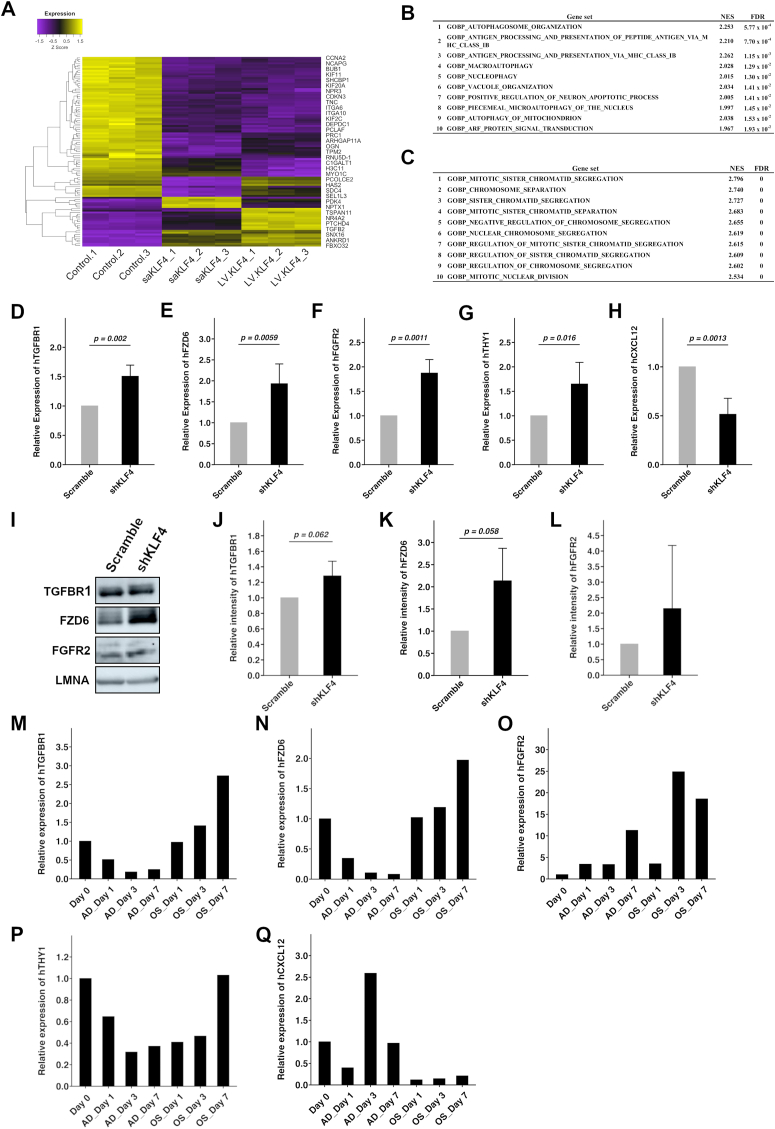
KLF4 regulates expression of signal receptor genes. (A) Heatmap of reanalyzed microarray data. (B) Result of GSEA analysis. Enriched gene sets represent upregulated genes in KLF4-OE MSCs. (C) Result of GSEA analysis. Enriched gene-sets represent downregulated genes in KLF4-OE MSCs. (D–H) Relative expressions of h*TGFBR1* (D), h*FZD6* (E), h*FGFR2* (F), h*THY1* (G) and h*CXCL12* (H) by qRT-PCR in KLF4-KD MEC. Five independent experiments were performed using five independent MEC clones. All quantitative data were normalized to those of each control cell. Error bars represent standard deviation. (I) Immunoblot of TGFBR1, FZD6 and FGFR2 in KLF4-KD and control cells. Lamin A (LMNA) was used as the internal control. (J–L) Measurement of immunoblot intensity in TGFBR1 (J), FZD6 (K) and FGFR2 (L). Experiments were performed on three independent clones. Error bars represent standard deviation. (M–Q) Relative expressions of h*TGFBR1* (M), h*FZD6* (N), h*FGFR2* (O), h*THY1* (P) and h*CXCL12* (Q) by qRT-PCR in adipogenic and osteogenic differentiation.

## 4 Discussion

MSCs are expected to form a useful cell source for use in regenerative medicine and in fact, about 1,100 clinical trials using MSCs have been registered over the last decade (http://www.clinicaltrials.gov). However, the heterogeneity of cells in a conventional population of MSCs has led to diversity in research results, making it difficult to understand the biological properties of MSCs. Besides, convincing markers used to define MSCs as "stem cells", such as *NANOG* and *OCT4* in pluripotent stem cells, remain unclear, making it difficult to maintain the quality of MSCs *in vitro*. Hence, identifying marker molecules to ensure the properties of MSCs are maintained is an important issue for clinical application.

In this study, we investigated marker molecules in our clonal MSC subtypes and focused on a transcription factor, *KLF4,* which was highly expressed in MEC. *KLF4* was initially identified as a growth arrest-associated factor which promotes expression of *CDKN1A* and represses cyclins such as *CCND1* and *CCNB1* [9]. Therefore, higher expression of *KLF4* in MEC compared with REC was a feasible observation. However, the expression level of *KLF4* was lower in SEC despite its slower proliferation rate in comparison with MEC. This result implies that *KLF4* retains another role other than negative regulation of the cell cycle in MSCs. Therefore, we investigated the relationship between the expression of KLF4 and the differentiation of MSCs.

Interestingly, KLF4 was significantly downregulated by 24 h after either adipogenic or osteogenic induction. Moreover, KLF4-KD in MECs not only enhanced cellular proliferation but also precipitated both formation of lipid droplets and calcium deposition, which occurred sooner in KLF4-KD in MECs than in controls. These data revealed that KLF4 functions as a regulator of proliferation and differentiation in MSCs. Next, based on reanalysis of microarray data in KLF4-OE MSCs, we identified a hundred three differentially expressed genes (27 genes upregulated and 76 downregulated) and confirmed upregulation of *TGFBR1*, *FGFR2*, *FZD6*, *THY1* and downregulation of *CXCL12* by KLF4-KD. Among upregulated genes, *TGFBR1*, *FGFR2* and *FZD6* encode the receptors for TGF-β, FGF and WNT signaling, respectively. These signal pathways have been reported to contribute to adipogenic and osteogenic differentiation in MSCs [[13], [14], [15], [16], [17]]. However, the significant increase of receptor proteins was not observed in KLF4-KD MECs even though the transcripts were significantly increased. This observation suggests that the expressions of those signal receptors are mediated by post-transcriptional regulation such as miRNA rather than transcriptional regulation by KLF4. Interestingly, knockdown of Dicer, an important RNA endonuclease in miRNA biosynthesis, in human bone marrow-derived MSCs has been shown to reduce osteogenic differentiation efficiency [18]. These findings show the importance of post-transcriptional regulation in the differentiation of MSCs and that the signal pathways via TGFBR1, FZD6 and FGFR2 are controlled by both transcriptional and post-transcriptional regulation. It is noteworthy that upregulation of receptors does not necessarily lead directly to activation of signal pathways. Therefore, it is suggested that KLF4 mediates susceptibility to environmental stimuli by regulating the transcription of these receptor genes. On the other hand, THY1, also known as CD90, is one of the cell surface markers included among the minimum criteria to define MSCs proposed by the International Society for Cell & Gene Therapy (ISCT) [1]. Moraes et al. reported that knockdown of THY1 led to enhanced adipogenic and osteogenic differentiation in human MSCs *in vitro* [19]. At a glance, their data are inconsistent with our data from KLF4-KD MEC, but they are consistent with our data showing that THY1 expression was reduced during adipogenic and osteogenic differentiation. CXCL12 is a chemokine ligand for its receptor CXCR4 and is essential for the maintenance of hematopoietic stem cells in the bone marrow [20]. Its deletion in a PDGFα^+^SCA1^+^ cell population, which is enriched for MSCs, resulted in reduced repopulation activity and quiescence of hematopoietic stem cells [21]. Although the impact of its expression on the characteristics of MSCs remains unknown, it is believed that MSCs have hematopoietic support ability [22]. Our data, showing that the expression of *CXCL12* was reduced after 24 h of adipogenic or osteogenic induction, may imply a relationship between the stemness of MSCs and hematopoietic support ability. Thus, our data support the hypothesis that KLF4 regulates adipogenic and osteogenic commitment in MSCs. Taken together, based on these findings and our previous work [6], our MSC subtypes can be classified as follows: MEC retains higher stemness than REC and SEC, while REC and SEC are committed to the adipogenic and osteogenic lineages, respectively. In addition, SEC is assumed to be in a more committed state due to its low proliferation ability. However, it is noted that more detailed studies will be required to validate this classification.

The mechanism of stemness maintenance by KLF4 differs between MSCs and pluripotent stem cells. In murine embryonic stem cells, KLF4 forms a complex with Nanog, Pou5f1 and Sox2 in the nucleus and prevents differentiation by promoting the expression of Nanog [23,24]. In contrast, the expression of KLF4 clearly differed between MEC and REC or SEC, but no difference in the expression of *NANOG* was observed in our microarray data. This observation suggests that there is another mechanism by which KLF4 prevents differentiation of MSCs. In addition, the existence of other genes regulating the differentiation of MSCs is not excluded. In fact, although our MSC clones were isolated from CD90/CD271 double-positive cells, expression of CD271 is almost abolished in established clones [6]. It is reported that CD271-positive cells isolated from human bone marrow and deciduous dental pulp cells have higher CFU-F activity and differentiation capacity compared with CD271-negative cells [25,26]. In addition, Sezaki et al. reported that the CD271^+^CD51^+^ population from human bone marrow has high colony forming capacity from a single cell [27]. As in our CD271^+^CD90^+^ population, their CD271^+^CD51^+^ population also exhibited clonogenic capacity. These facts strongly suggest that CD271 contributes to maintaining the stemness of MSCs, although further studies will be required to clarify the mechanism.

In summary, we showed that homogeneous MSCs are advantageous for studying the biological properties of MSCs and revealed that KLF4 plays a role in regulating the proliferation and the commitment to adipogenic and osteogenic lineages of progenitors in the bone marrow-derived MSC population. We believe that accumulating research using homogeneous MSCs would lead to more reliable findings and more consistent effects in future clinical applications.

## CRediT authorship contribution statement

**Kenichi Miyamoto:** Writing – original draft, Project administration, Methodology, Investigation, Funding acquisition, Data curation, Conceptualization. **Satoru Miyagi:** Supervision, Resources, Methodology. **Rintaro Yoshikawa:** Supervision. **Yuichi Michikawa:** Funding acquisition. **Yumi Matsuzaki:** Supervision, Project administration.

## Funding

This work was supported by JSPS KAKENHI [18K07846] and in part by an Intramural Research Grant from the National Institute for Quantum Science and Technology.

## Declaration of competing interest

Yumi Matsuzaki is the CSO of PuREC Co., Ltd.

## Figures and Tables

**figs1:**
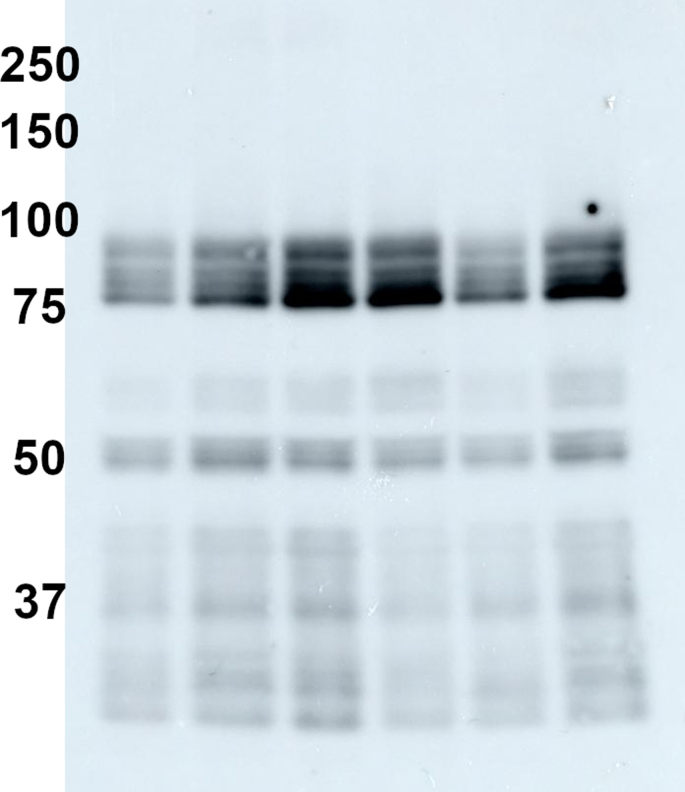


**figs2:**
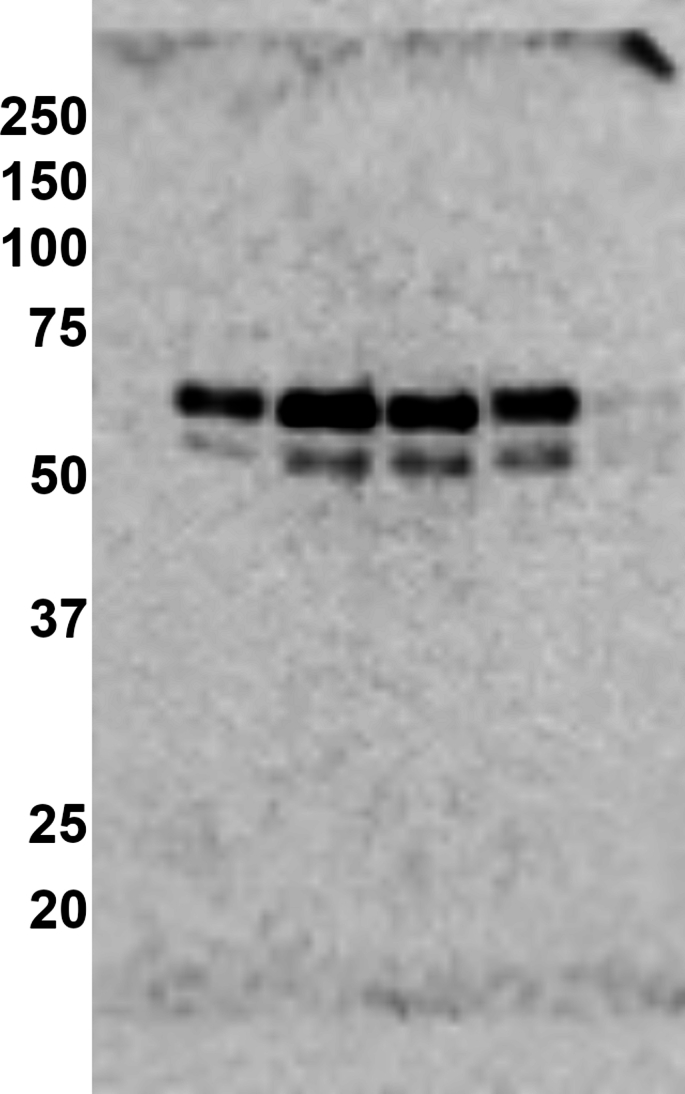


**figs3:**
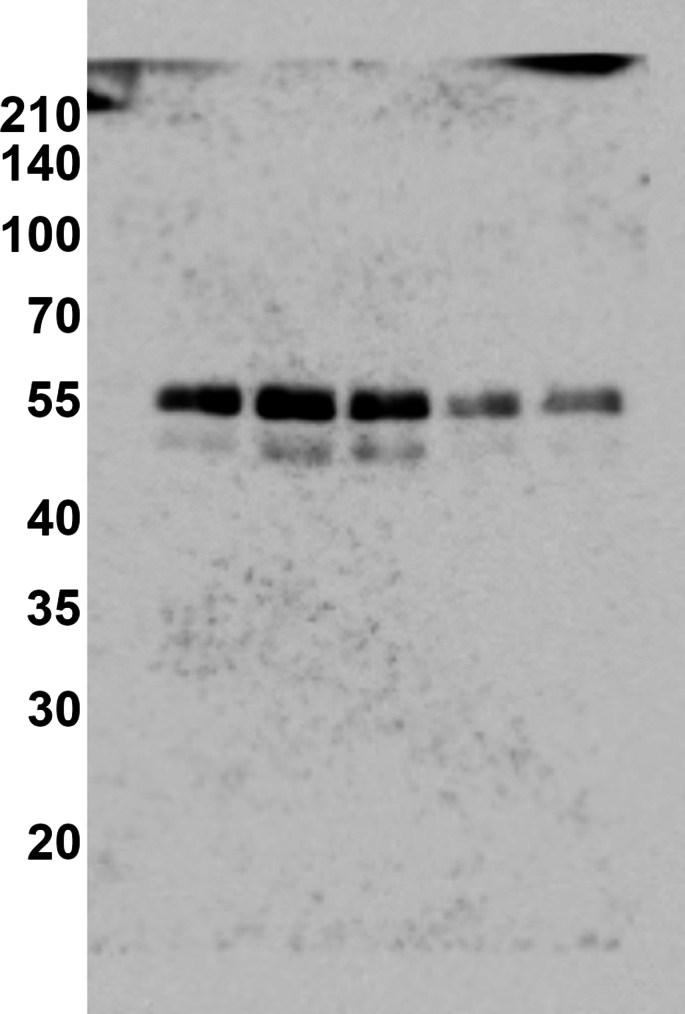


**figs4:**
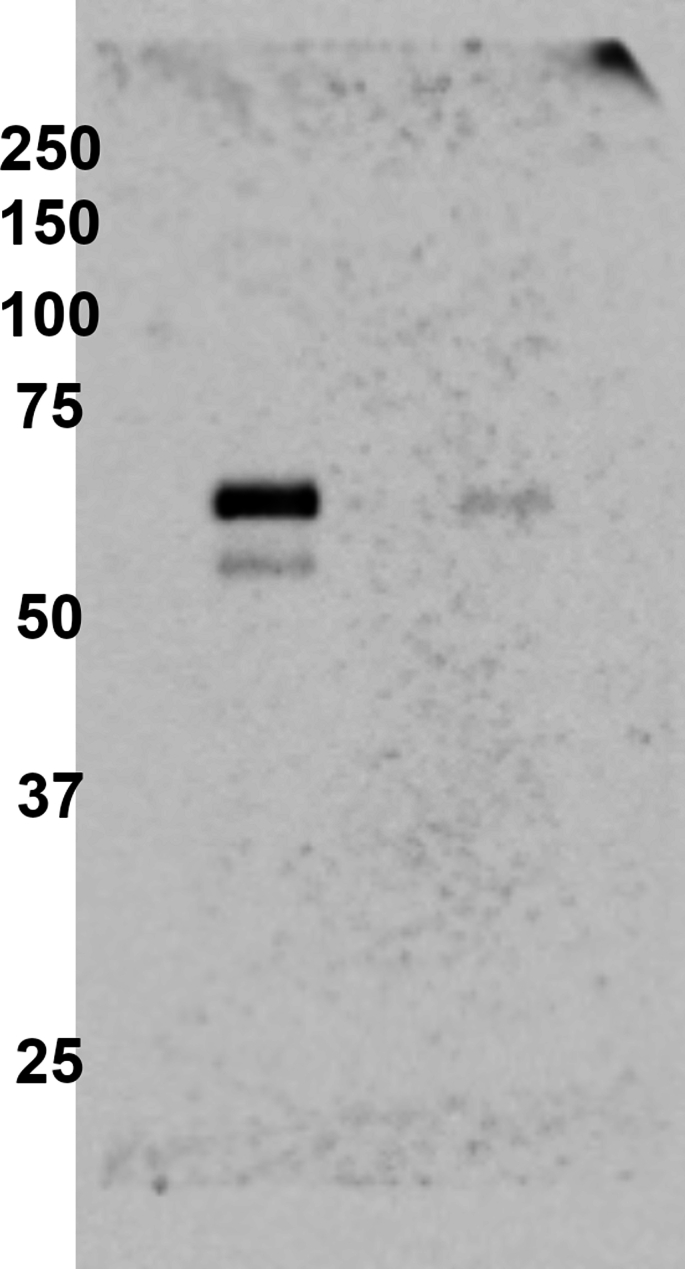


**figs5:**
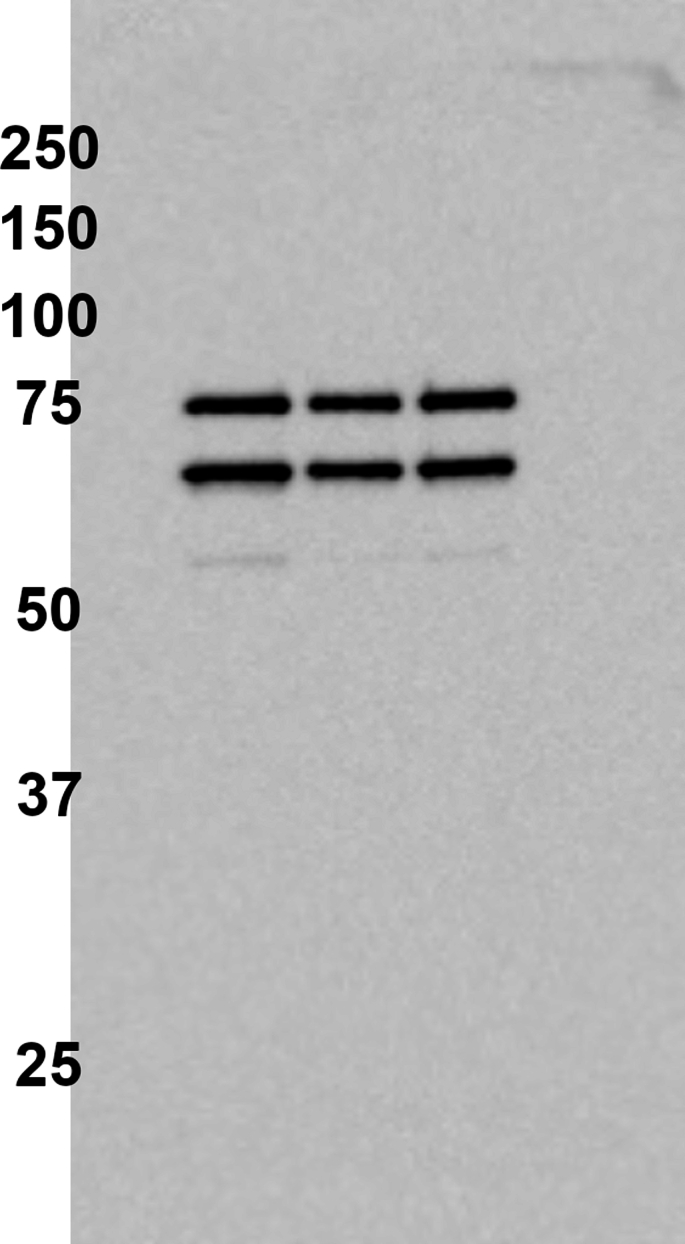


**figs6:**
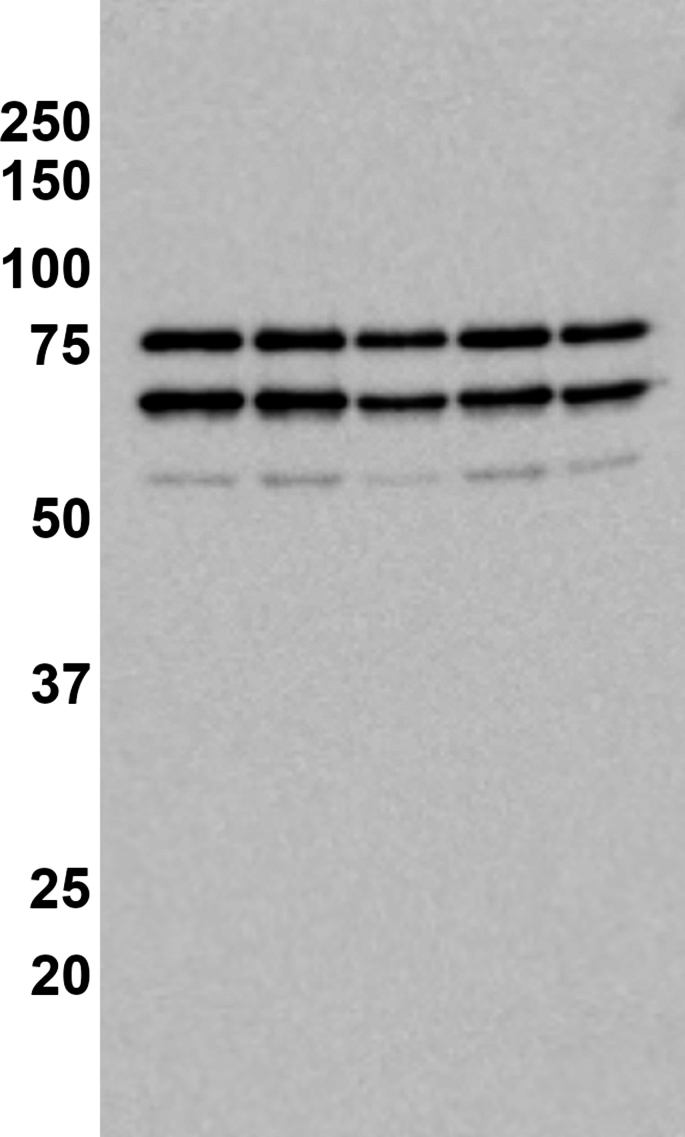


**figs7:**
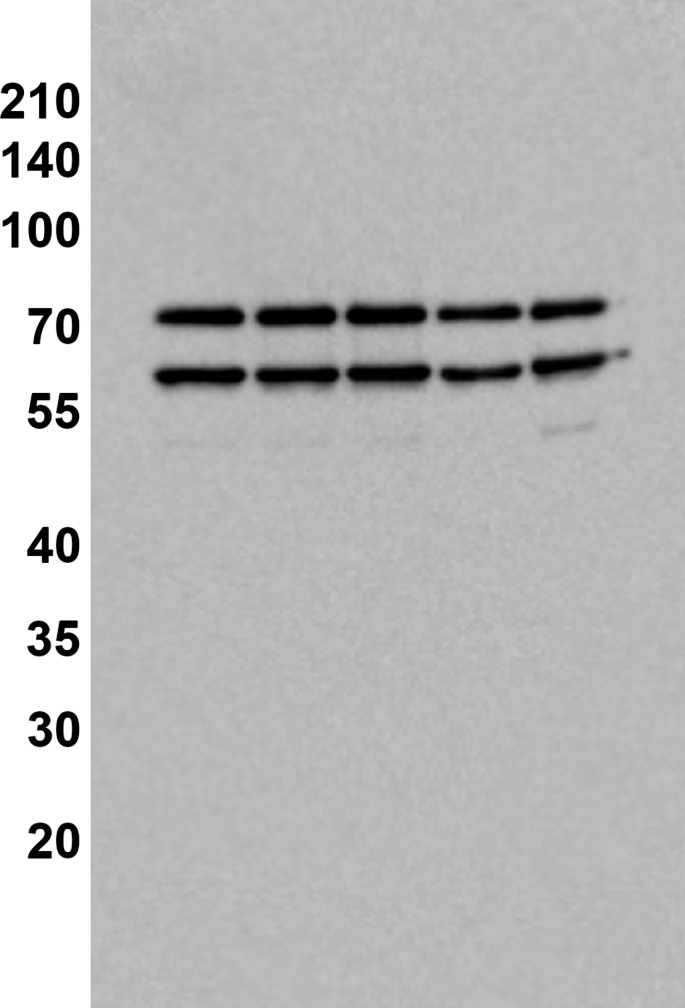


**figs8:**
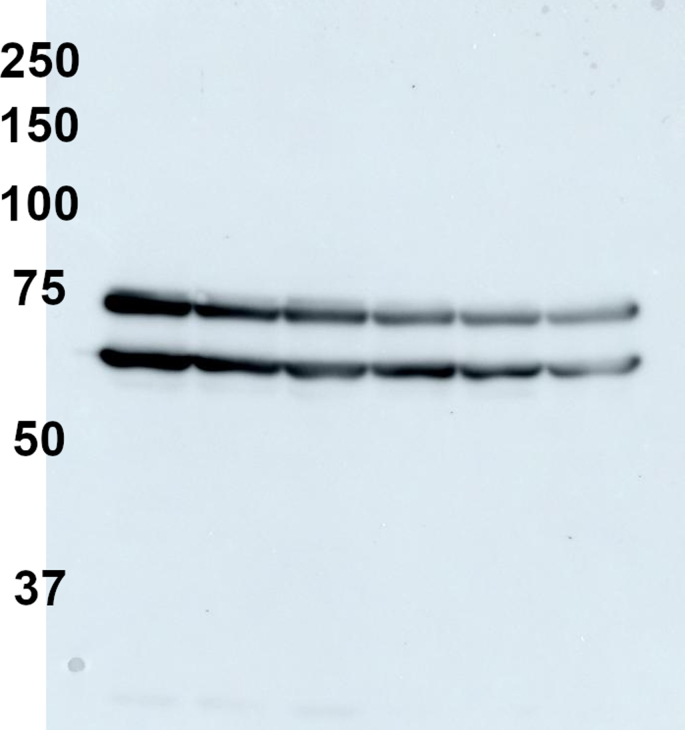


**figs9:**
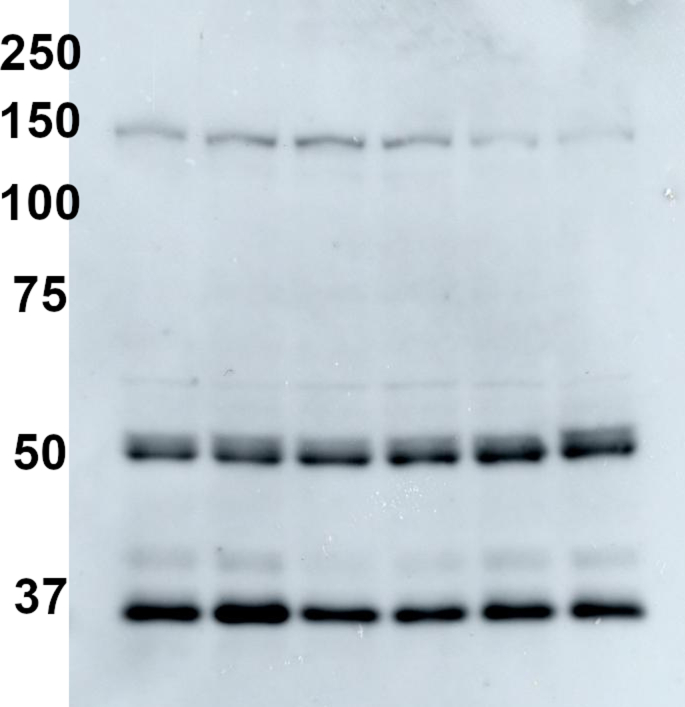


**figs10:**
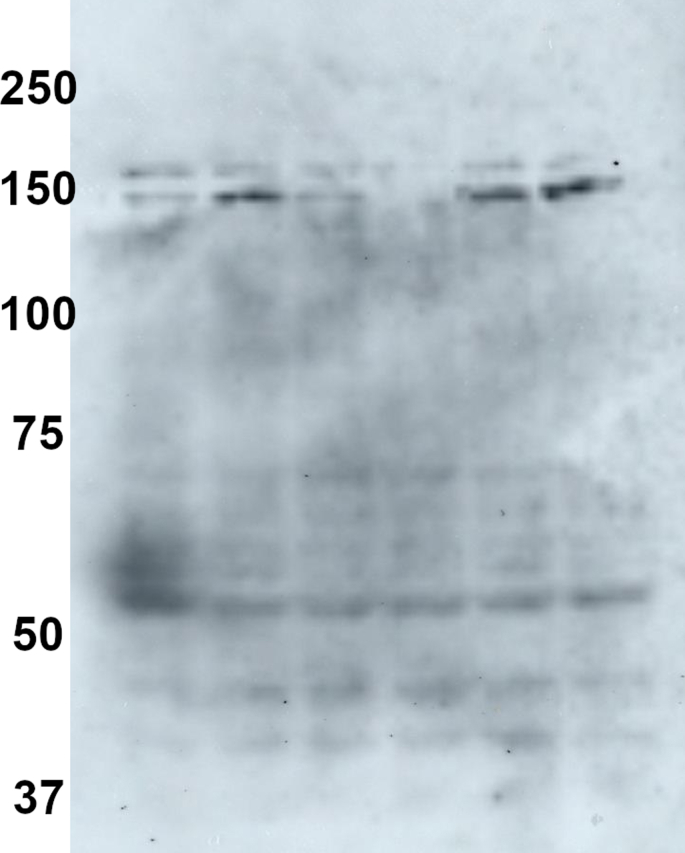


## Data Availability

Data will be made available on request.
