# Supplementary material for: Krüppel-like factor 4 regulates cellular proliferation and differentiation in human bone marrow-derived mesenchymal stem cells
Source: Biochem Biophys Rep. 2025 Sep 6;44:102241. doi: 10.1016/j.bbrep.2025.102241 (PMC12447919; doi:10.1016/j.bbrep.2025.102241)
Supplement: Multimedia component 2 [file mmc2.docx]

**Supplemental Table 1. Primer list used for qRT-PCR**

| **Target Gene** | **Direction** | **Sequence (5’ – 3’)** |
| --- | --- | --- |
| h*KLF4* | Forward | ACAAAGAGTTCCCATCTCAAGG |
|  | Reverse | TACGGTAGTGCCTGGTCAGTT |
| h*PPARG* | Forward | GCGATTCCTTCACTGATACACTGTC |
|  | Reverse | ATTACGGAGAGATCCACGGAGC |
| h*RUNX2* | Forward | CATAACCGTCTTCACAAATCCTC |
|  | Reverse | GAGTCATCAAGCTTCTGTCTGTG |
| h*FGFR2* | Forward | TCCAGAAGCCCTGTTTGATAGA |
|  | Reverse | TGAAGATCTCCCACATTAACACC |
| h*FZD6* | Forward | AATTGTTGGCATCTCTGCTGT |
|  | Reverse | TACTCTTCGACTTTCACTGATTGG |
| h*MKI67* | Forward | TACAAGACTCGGTCCCTGAAA |
|  | Reverse | GTTATTTGCTGTTCTGCCTCAGT |
| h*TGFBR1* | Forward | AAATTGCTCGACGATGTTCC |
|  | Reverse | CTGATGGGTCAGAAGGTACAAGA |
| h*THY1* | Forward | CTACTTATCCGCCTTCACTAGCA |
|  | Reverse | TGATGCCCTCACACTTGACC |
| h*CXCL12* | Forward | GAGCCAACGTCAAGCATCTC |
|  | Reverse | CCAGGTACTCCTGAATCCACTTT |
| h*EEF1A1* | Forward | GCTGCCATTGTTGATATGGTT |
|  | Reverse | CTTCTTGTCCACTGCTTTGATG |
