# Supplementary material for: Krüppel-like factor 4 regulates cellular proliferation and differentiation in human bone marrow-derived mesenchymal stem cells
Source: Biochem Biophys Rep. 2025 Sep 6;44:102241. doi: 10.1016/j.bbrep.2025.102241 (PMC12447919; doi:10.1016/j.bbrep.2025.102241)
Supplement: Multimedia component 3 [file mmc3.docx]

**Supplemental Table 2. Fold change and FDR of down-regulated genes in KLF4-OE MSC**

| **Gene** | **log_2_FC** | **FDR** |
| --- | --- | --- |
| *TGFBR1* | -2.395 | 1.63x10^-5^ |
| *FZD6* | -1.696 | 9.19x10^-4^ |
| *THY1* | -1.353 | 4.34x10^-3^ |
| *CXCL12* | -0.851 | 3.96x10^-2^ |
| *FGFR2* | -0.664 | 8.68x10^-2^ |

Fold change and FDR of selected genes from reanalyzed microarray data by Voutila et al. [11].
